# Effect of Zonal Laser Texturing on Friction Reduction of Steel Elements in Lubricated Reciprocating Motion

**DOI:** 10.3390/ma17102401

**Published:** 2024-05-16

**Authors:** Slawomir Wos, Waldemar Koszela, Andrzej Dzierwa, Pawel Pawlus

**Affiliations:** The Faculty of Mechanical Engineering and Aeronautics, Rzeszow University of Technology, al. Powstancow Warszawy 12, 35-959 Rzeszów, Poland; wosslawomir@prz.edu.pl (S.W.); adzierwa@prz.edu.pl (A.D.)

**Keywords:** surface texturing, reciprocation motion, coefficient of friction

## Abstract

During co-action between contact elements in reciprocating motion, different working conditions exist in outer and inner zones of stationary elements. Because the tribological effects of surface texturing depend on the operating conditions, various dimple patterns were created in the middle part of the steel disc and near the reversal points. The behaviors of variable dimple patterns were compared with those of uniform texturing and untexturing. It was found that the dimple patterns in the middle disc zone depended on the resistance to motion. The best tribological behavior was obtained for a pit area ratio of 13% and diameter of 0.4 mm in the inner zone, and pit area ratio of 3% and diameter of 0.2 mm in the outer zones. Low resistance to motion and the smallest friction variation of all tested sliding pairs were achieved. For the same pit area ratio of 13% in a disc of 0.4 mm, the dimple diameter behaved better than in the 0.2 mm diameter disc. The greatest decrease in the coefficient of friction of 85% compared to untextured sliding pair was achieved for uniform laser texturing with a pit area ratio of 13% and dimple diameter of 0.4 mm, when the normal load was 40 N and frequency of displacement was 20 Hz.

## 1. Introduction

Surface texturing is a method leading to an improvement in tribological properties of assembly by creating dimples on sliding surfaces. These dimples may cause a decrease in friction. Surface texturing can also be used to increase the seizure and abrasive wear resistances. 

This technique is very popular, and applications of surface texturing have been described in recent reviews [1,2,3,4,5]. Rosenkranz et al. [1] discussed the tribological effects of surface texturing in rolling and sliding contacts. Nsilani Kouediatouka [2] discussed the creation of dimples by laser texturing with tribological applications. Gachot et al. [3] presented positive and negative tribological effects of surface texturing in different lubrication regimes. Lu and Wood [4] presented the tribological behavior of surface texturing in the following applications: piston-ring and cylinder liners, cutting tools, sealing and journal bearings. Mao et al. [5] reviewed the recent advances in laser surface texturing for the improvement of tribological properties of engineering materials. Pawlus et al. [6] discussed the impacts of surface texturing on cylinder liners.

There are many texturing techniques, including burnishing [7,8] and abrasive jet machining [9,10]. However, laser texturing is the most widely used technique [11,12]. Textured surfaces are usually described by pit area ratio and dimple sizes. Typically, circular dimples are employed; they are characterized by depth and diameter. The ratios between the depth and diameter of the circular dimples are between 0.01 and 0.1 [11].

The application of surface texturing depends on the operating conditions [13]. Typically, it is utilized in the sliding unidirectional contact. Research works on reciprocating motion are rarely carried out. The conditions of reciprocating sliding are different in various zones of lubricated contact; near reversals, boundary lubrication occurs, but in the middle part of the stroke, hydrodynamic lubrication exists. 

Costa and Hutching [14] studied the effects of various shapes of oil pockets on oil film thickness growth in hydrodynamic lubrication under reciprocation motion. The highest increase was achieved for chevrons, and the smallest for grooves. The authors of the papers [15,16,17,18,19,20] studied the effects of the shape of the oil pockets on friction reduction in reciprocating motion using test rigs. Podgornik et al. [15] revealed that grooves typically caused greater friction under the starved lubrication condition. Saeidi et al. [16] obtained a reduction in friction for oval oil pockets positioned perpendicularly to the movement direction in conditions close to hydrodynamic lubrication. 

Lu et al. found that square [17] and triangular pockets [18] caused a decrease in friction in line contact conditions. In [19], they tested samples with square flat, square slope and triangular flat oil pockets, and derived beneficial effects from the anisotropic textures in all lubrication regimes.

Wos et al. [20] observed the beneficial effect of sandglass-shaped oil pockets, especially with a small pit area ratio. Vilhena et al. [21] achieved a reduction in friction thanks to surface texturing in reciprocating sliding at lower speeds; opposing effects were obtained at higher speeds. 

The contact between the piston ring and the cylinder bore is a common example of coaction in a lubricated reciprocating regime. Ryk et al., using flat samples [22] and piston rings and cylinder liner segments [23], revealed that the partial texturing of the surfaces of the piston ring provided more friction reduction than full surface texturing [24]. 

Surface texturing was also applied to the surfaces of the cylinder liners. Plateau honing was probably the first example of surface texturing of cylinder bores. However, the dimples were also created on the surfaces of the cylinders. Duffet et al. [25] obtained a longer life and a lower friction due to oil pocket creation on the cylinder surface. Fan and Zhong [26] found that oil pockets on the liner surface caused increased seizure resistance. Zhan and Yan studied the effects of the array of dimples presented on cylinder wall surfaces on wear reduction under starved [27] and full lubrication [28] conditions. They obtained greater reductions in cylinder wear under full lubrication compared to starved lubrication due to surface texturing [29]. Morris et al. [30] analyzed the effects of various chevron patterns on the decrease in friction. Grabon et al. [31] achieved a friction reduction of up to 50% due to additional dimples created on honed cylinder surfaces under conditions of good lubrication in reciprocating sliding. Vladescu et al. [32] revealed that grooves normal to the sliding direction caused a reduction in friction in contrast to grooves along the sliding direction, where friction reduction was caused by the increase in oil film thickness [33]. Yousfi et al. [34] reduced friction by creating ellipses perpendicular to the sliding direction; this reduction was greater than that obtained after helical slide honing. Rao et al. [35] found that thread grooves created on the liner surface reduced wear. Miao et al. [36] achieved a reduction in friction by texturing both surfaces from piston ring–cylinder liner assembly. 

The presented works addressed the tribological effects of single-scale textures. In reciprocating sliding, the surface texture fulfills multiple functions in different friction regimes. The use of multi-scale surface texturing [37] seems to be a promising approach.

Zhu et al. [38] found that in reciprocating sliding, a higher pit area ratio at the reversal points of a block simulating a cylinder liner surface and a smaller one in the middle liner part caused friction reductions, while the opposite design caused an increase in friction. 

Vladescu et al. [39] obtained similar findings. In the boundary lubrication regime, the pit area ratio on the liner surface must be large, and dimples should be deep and wide to increase the oil retention volume. However, at the transition between mixed and hydrodynamic lubrication, the pit area ratio of narrow and shallow oil pockets should be smaller. The dimple patterns should vary along the stroke. Surface texturing in the regime of full lubrication is not recommended.

The presented results are in agreement regarding the application of additional dimples near reversal points of a cylinder liner. However, Urabe et al. [40] machined dimples at the midpoint; the pit area ratio was large, at 50%, and dimple depth was small, at 2 µm. A friction decrease was achieved by reducing the real area of contact, which led to oil film thickness reduction, in contrast to creating oil pockets near reversals. These results correspond to those of Hsu et al. [41], who recommended wide and shallow dimples in a full lubrication regime and narrow and deep dimples in mixed and boundary friction.

Zhou et al. [42] analytically studied the influence of patterns of oil pockets on the increase in the oil film thickness. The greatest growth in film thickness was achieved under variable texturing, depending on the increasing pit area ratio as speed decreased (near reverse point). Oil film thickness was also predicted for the smallest dimple density on the entire cylinder’s texture.

The texturing of the entire surface of the cylinder liner is connected with a growth in the oil consumption. Ergen et al. [43] obtained smaller oil consumption by creating oval oil pockets only near the reverse point. The authors of the articles [44,45] obtained similar findings. Hua et al. [44] studied the effect of discriminating partition laser texturing on engine performance. Kang et al. [45] textured various cylinder regions. Partial surface texturing caused a lower oil consumption and blow-by than the texturing of the entire cylinder liner surface. However, surface texturing also caused a reduction in the fuel consumption. Then, the optimal pattern of the oil pockets should be searched to decrease the fuel and oil consumptions. In [43,44,45], the pit area ratio near the top dead center was higher than in the middle part of the cylinder liner surface. Wos et al. [46] found that friction reduction due to surface texturing was larger at smaller temperatures, compared to work at higher temperatures.

The results regarding the influence of variable dimple arrays during lubricated reciprocating sliding on friction reduction were conflicting. Flexible arrays of oil pockets were applied mainly to simulate the co-action in the piston ring–cylinder liner assembly. The aim of this work is to study the influence of zonal laser texturing on friction in lubricated reciprocating sliding during the contact of steel samples in a ring shape under various operating conditions. The effects of zonal texturing on friction reduction for this type of contact have not been reported in the technical literature. Therefore, this research is innovative.

## 2. Materials and Methods

**Figure 1:**
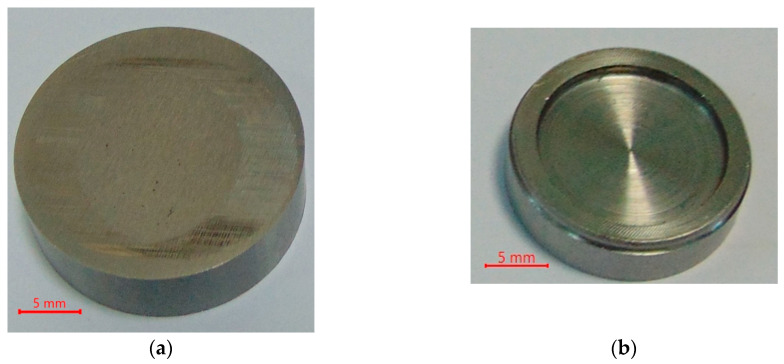
Photos of the untextured sample (**a**) and the counter-sample (**b**).

**Figure 2:**
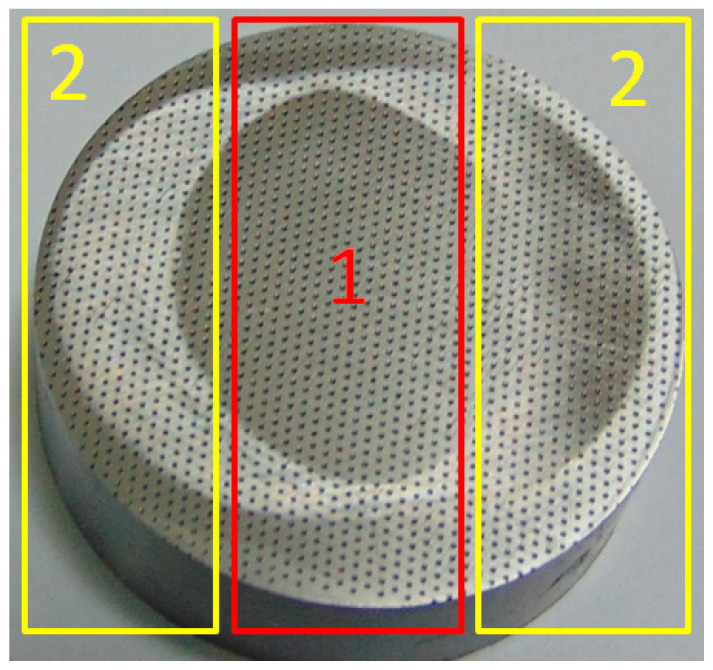
Wear scar view with marked coaction zones between sample and counter-sample: inner zone (1) and outer zones (2).

The experiments were carried out in lubricated conformal reciprocating motion using the Optimol SRV5 tester. The contact region had the shape of a ring. The lower disc was the sample. It had 7.9 mm thickness and 25 mm diameter. The upper disc was a counter-sample and had 5 mm thickness and 18 mm diameter—Figure 1. Both discs were prepared from 42CrMo4 steel of 44 ± 2 HRC hardness. The steel composition excluded iron: 0.38–0.45% of carbon, 0.6–0.9% of manganese, 0.9–1.2% of chrome and 0.15–0.3% of molybdenum. Only samples (lower discs) were textured. Before texturing, the samples were subjected to grinding. They achieved an average roughness height (Ra parameter) of 0.1–0.15 µm. Surfaces from lower disc were laser-textured to obtain dimples in entire disc areas (uniform texturing) and various dimple arrays in different zones (zonal texturing). The influence of partial sample texturing was also studied. Figure 2 shows the scheme of coaction between elements of the sliding pair.

In the inner zone (1) (Figure 2), continuous contact occurred between the sliding elements; the space above the dimples was closed. In outer zones (2), discontinuous contact between the sample and the counter-sample developed; the space above the dimples was periodically opened and closed. 

Surface texturing was performed by a laser engraver SpeedMarker 300, made by Trotec^®^ (Marchtrenk, Austria). The laser power was 20 W, the focal diameter and length were 64 µm and 254 mm, respectively, the pulse duration was 1.5 ns, the marking speed was 200 mm/s, and the pulse repetition rate was 820 kHz. 

**Figure 3:**
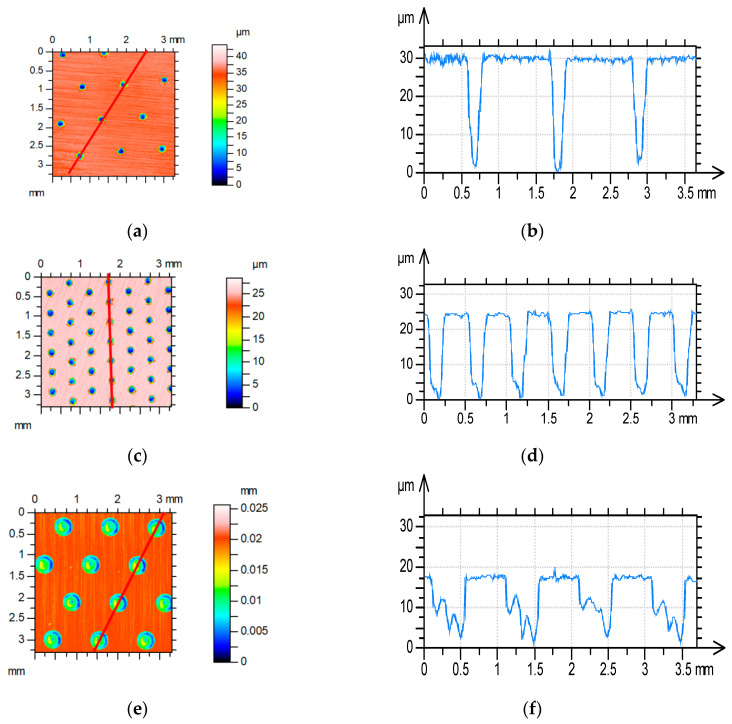
Types of disc laser texturing: T_02,3_ (**a**,**b**), T_02,13_ (**c**,**d**), T_04,13_ (**e**,**f**), contour plots (**a**–**c**), profiles (**c**–**e**).

Three types of textured surfaces were tested. The dimples had circular shapes. The dimple depths were 25 ± 5 µm. These types were described by dimple diameter and pit area ratio. Type T_04,13_ of the textured surface was characterized by a dimple diameter of 0.4 mm and a pit area ratio of 13%, T_02,13_ by a dimple diameter of 0.2 mm and a pit area ratio of 13%, and T_02,3_ by a dimple diameter of 0.2 mm and a pit area ratio of 3%. T_unt_ refers to the untextured type. The sizes and distributions of oil pockets were selected due to their ability to catch wear products and to produce hydrodynamic lift by changing the pressure in the oil film. Figure 3 presents examples of textured disc types.

Table 1 presents the tested disc surfaces. The surfaces S_I-04,13_ and S_O-04,13_ were partially textured in the inner and outer zones, respectively, and in both cases the dimple diameter was 0.4 mm and the pit area ratio was 13%. Surfaces S_04,13_ and S_02,13_ were uniformly textured, the pit area ratio was 13% and the dimple diameters were 0.4 and 0.2 mm, respectively. Untextured surface S_unt_ was also tested. The other surfaces listed in Table 1 (S_I-04,13,O-02,3_, S_I-02,13,O-04,13_, S_I-02,3,O-04,13_, and S_I-02,13,O-02,3_) had different dimple patterns in the outer and inner zones. Surface S_I-04,13,O-02,3_ was characterized by a dimple diameter of 0.4 mm and a pit area ratio of 13% in the inner zone, and a dimple diameter of 0.2 mm and a pit area ratio of 3% in the outer zones; surface S_I-02,13,O-04,13_ was characterized by a dimple diameter of 0.2 mm and a pit area ratio of 13% in the inner zone and a dimple diameter of 0.4 mm and pit area ratio of 13% in the outer zones; surface S_I-02,3,O-04,13_ was characterized by a dimple diameter of 0.2 mm and a pit area ratio of 3% in the inner zone and a dimple diameter of 0.4 mm and a pit area ratio of 13% in the outer zones, and surface S_I-02,13,O-02,3_ was characterized by a dimple diameter of 0.2 mm and a pit area ratio of 13% in the inner zone and a dimple diameter of 0.2 mm and a pit area ratio of 3% in the outer zones.

**Figure 4:**
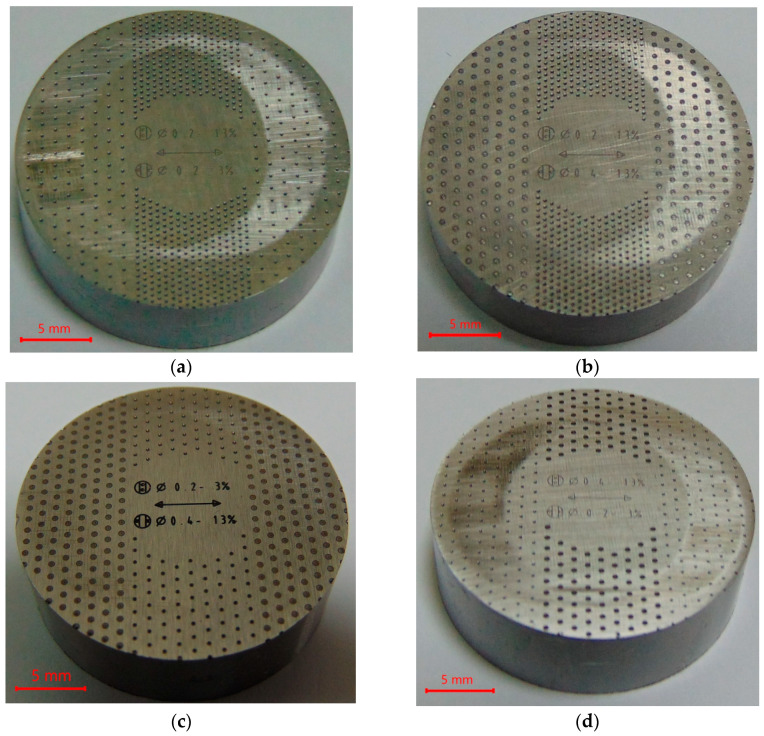

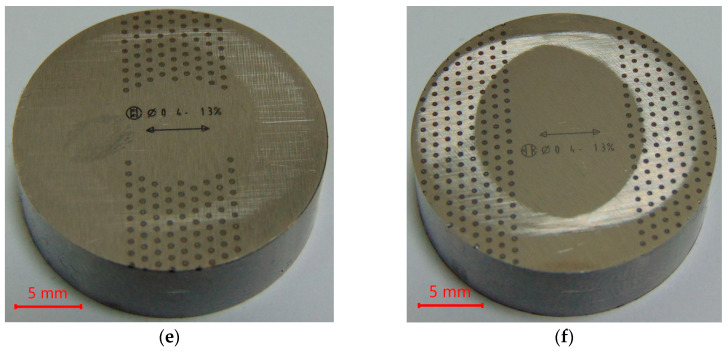
Photos of selected disc samples after tribologic tests: S_I-02,13,O-02,3_ (**a**), S_I-02,13,O-04_,_13_ (**b**), S_I-02,3,O-04,13_ (**c**), S_I-04,13,O-02,3_ (**d**), S_I-04,13_ (**e**), S_O-04,13_ (**f**).

Figure 4 shows photos of selected samples after tests with signs of wear.

A stroke of 3 mm and a temperature of 30 °C were the constant parameters of the tribological tests. The temperature was selected based on previous research [46], in which the friction decrease due to texturing was greater compared to the work at higher temperatures. Normal loads of 40 and 80 N and frequencies of displacement of 20 and 40 Hz were the variable test parameters. The investigations were carried out stepwise, with each subtest duration being 5 min. Each tribological test was repeated 3 times. Before each test, one drop of mineral oil L-AN-46 (approximately 0.08 ± 0.01 mL) was applied to the contact area. The kinematic viscosity of this oil at 40 °C was 46.0 mm^2^/s, the kinematic viscosity at 100 °C was 6.66 mm^2^/s, and the viscosity index was 96. This was utilized in previous works of the authors of this paper, including [43]. This oil was chosen because it had a low number of additives. Before and after wear tests, measurements of surface topographies of the lower discs were performed using the Talysurf CCI Lite optical profilometer (white light interferometer). The objective 5× was used. Non-measured points were filled up, and data were only leveled. Images of the tested disc surfaces were obtained with the Phenom ProX scanning electron microscope (SEM) produced by ThermoFisher Scientific from Waltham, MA, USA.

## 3. Results and Discussion

**Figure 5:**
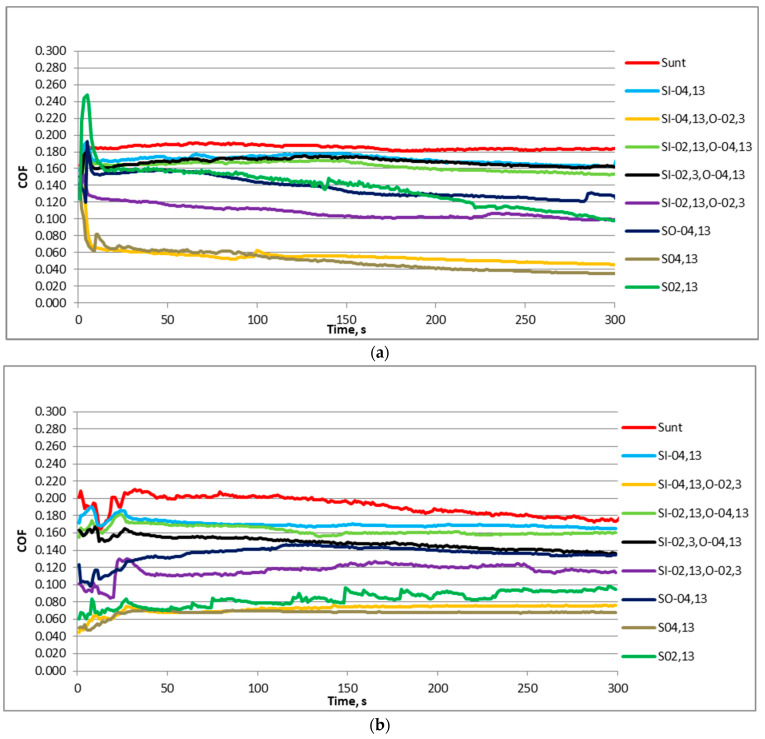
Representative courses of the coefficient of friction versus time for all sliding pairs for the normal force of 40 N and the frequency of displacement of 20 (**a**) and 40 Hz (**b**).

Figure 5 shows representative plots of the friction coefficient in time for the displacement frequencies of 20 Hz and 40 Hz and the normal load of 40 N of various sliding pairs. When the frequency of displacement was lower (Figure 5a), the initial fluctuations in the friction coefficient happened for the first 5 s. Then, the friction coefficient abruptly decreased, and then typically decreased. However, in some cases, after the initial fluctuation, the friction behaved differently; it could increase and decrease, or it increased. Different plots of the friction coefficient were shown for higher frequency of displacement (Figure 5b), especially in the initial phases of the tests. When the frequency of displacement was 40 Hz, the coefficients of friction initially increased, obtaining the highest values after 30–35 s, and then they decreased, and typically obtained stable values after 75–150 s; however, in some cases, fluctuations in the friction coefficient occurred. A high variability in the coefficient of friction for a sliding pair with sample S_02,13_ was seen for both frequencies of displacement.

**Figure 6:**
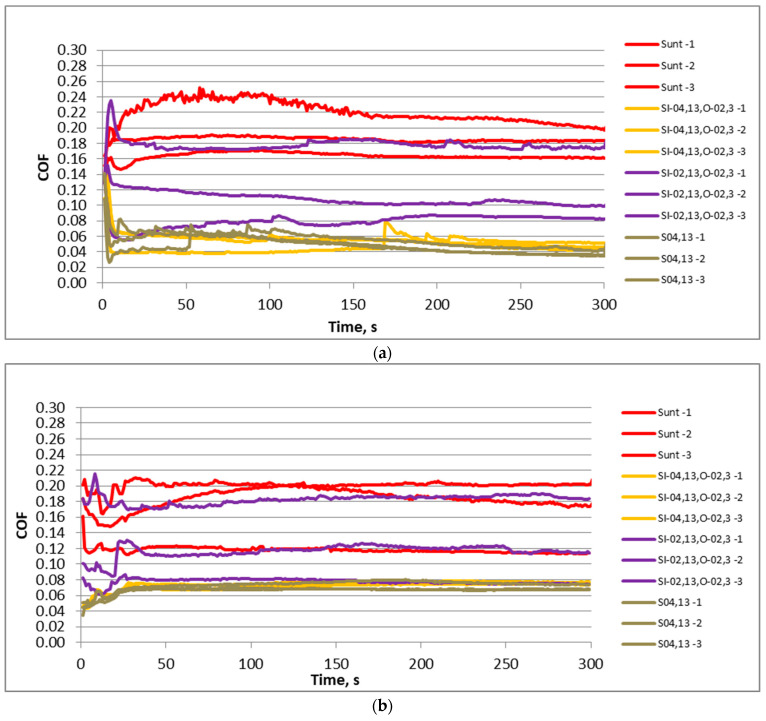
The friction coefficient versus time for selected sliding pairs with samples S_unt_, S_I-02,13,O-02,3_, S_I-04,13,O-02,3_ and S_04,13_ for the normal force of 40 N and the displacement frequency of 20 (**a**) and 40 Hz (**b**).

Figure 6 shows graphs of the friction coefficient versus time for three test repetitions of selected sliding pairs for the normal force of 40 N. Independently of the frequency of displacement, the untextured assembly caused the highest friction coefficient, with comparatively high variation. The sliding pair with the disc S_I-02,13,O-02,3_ led to smaller values and a slightly higher variation of friction coefficient than the untextured sliding pair. The lowest values and fluctuation of the friction coefficient due to test repetitions were achieved for assemblies with samples S_I-04,13,O-02,3_ and S_04,13_. These behaviors were found for both displacement frequencies.

**Figure 7:**
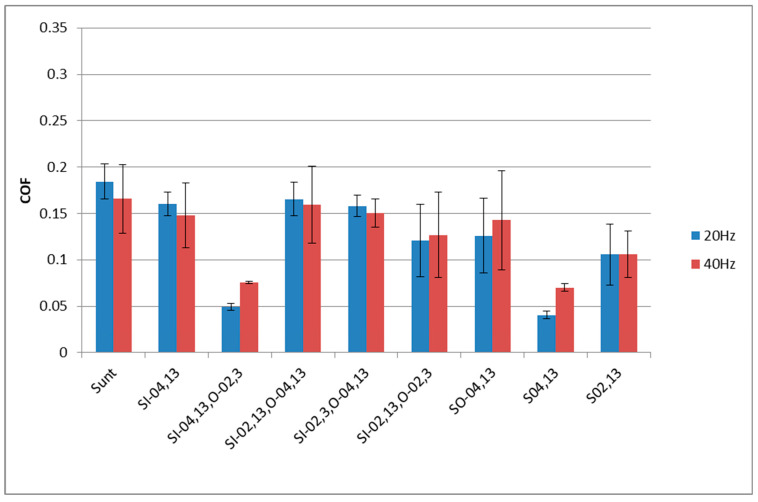
Average values and error bars of the friction coefficient of different assemblies tested at the normal load of 40 N for the last 100 s.

Figure 7 presents the mean values and error bars (standard deviations) of the coefficient of friction for sliding samples tested with the normal force of 40 N. Only stabilized friction coefficient values were considered, from the last 100 s of each test. 

When the frequency of displacement was 20 Hz, the largest coefficient of friction was obtained for the untextured assembly. A slightly smaller friction was found for assemblies containing samples S_I-02,13,O-04,13_ and S_O-04,13_. In the mentioned cases, the error bars overlapped, and the standard deviations were similar to each other. The presence of dimples caused a substantial reduction in the friction coefficient for the samples: S_I-02,13,O-02,3_, S_I-02,3,O-04,13_, and S_I-04,13_. The sample S_02,13_ led to further reductions in the friction coefficient; however, the variation in friction was large. The highest friction reduction of 85% was achieved for the disc sample S_04,13_. One can see that, for the same pit area ratio, the smaller dimple diameter produced higher resistance to motion. A slightly higher coefficient of friction was obtained for sample S_I-04,13,O-02,3._ In the last two cases mentioned (sliding pairs with samples S_04,13_ and S_I-04,13,O-02,3_), the variations in the friction coefficient due to test repetitions, visible in Figure 7, were very low.

Similar results were obtained when the frequency of oscillations increased to 40 Hz. In most cases, the variation in the friction coefficient increased compared to the operation at a lower frequency of displacement. The highest friction coefficient was obtained for the untextured sample S_unt_ followed by samples S_I-02,13,O-04,13_, S_I-04,13_, S_I-02,3,O-4,13_, S_I-02,13,O-02,3_ and S_O-04,13_. In these six cases, error bars of the coefficient of friction overlapped. The smallest friction occurred for sliding pairs containing samples S_04,13_ and S_I-04,13,O-02,3_. These assemblies were also characterized by low friction fluctuation due to repeated tests. The smallest friction variation was achieved by the sliding pair containing sample S_I-04,13,O-02,3_. The highest reduction was approximately 55%. Disc sample S_02,13_ led also to a significant reduction in the friction coefficient compared to an untextured sliding pair, of about 40%; however, the variation in the friction coefficient was great. 

**Figure 8:**
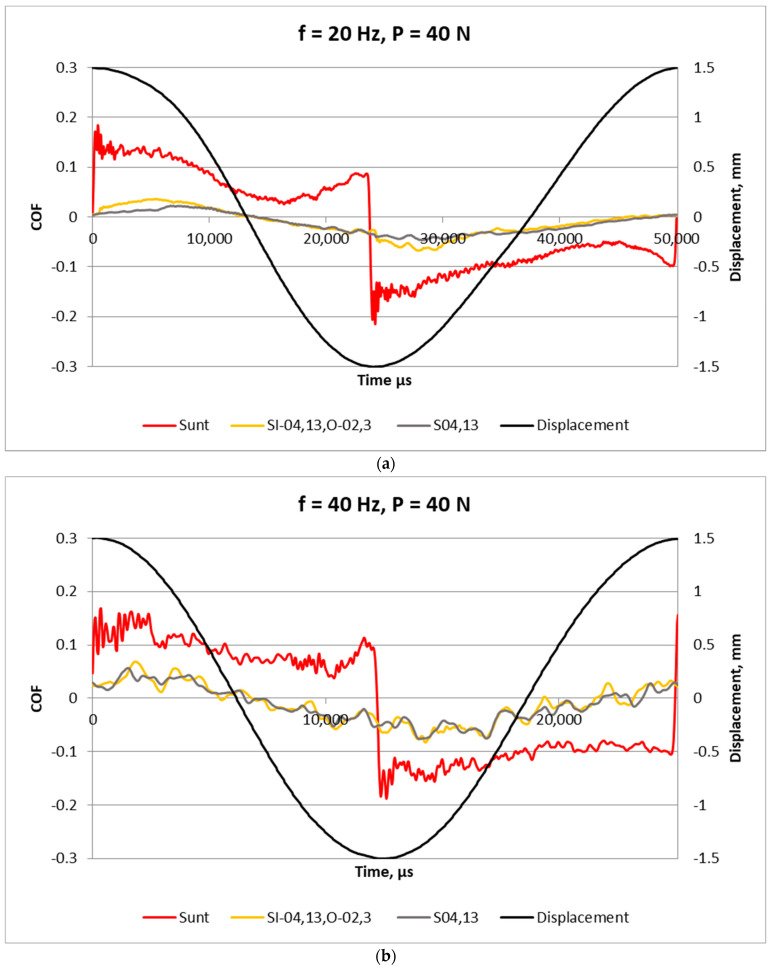
High-frequency analysis of the coefficients of friction obtained for untextured and textured samples for a normal force of 40 N and displacement frequency of 20 (**a**) and 40 Hz (**b**).

Figure 8 presents examples of the friction coefficient within one stroke for assemblies characterized by the smallest frictional resistance with samples S_I-04,13,0-02,3_ and S_04,13_, and the behavior of an untextured sliding pair is also shown. Both textured samples led to significant friction reduction compared to that of the untextured disc. The highest friction reduction occurred near the reversal points, where an oil film probably existed. In the middle zone, small friction probably led to hydrodynamic lubrication. The friction forces were more stable for smaller frequency of displacement (20 Hz); in these cases the friction reduction due to surface texturing was larger compared to performances at the higher frequency of 40 Hz.

**Figure 9:**
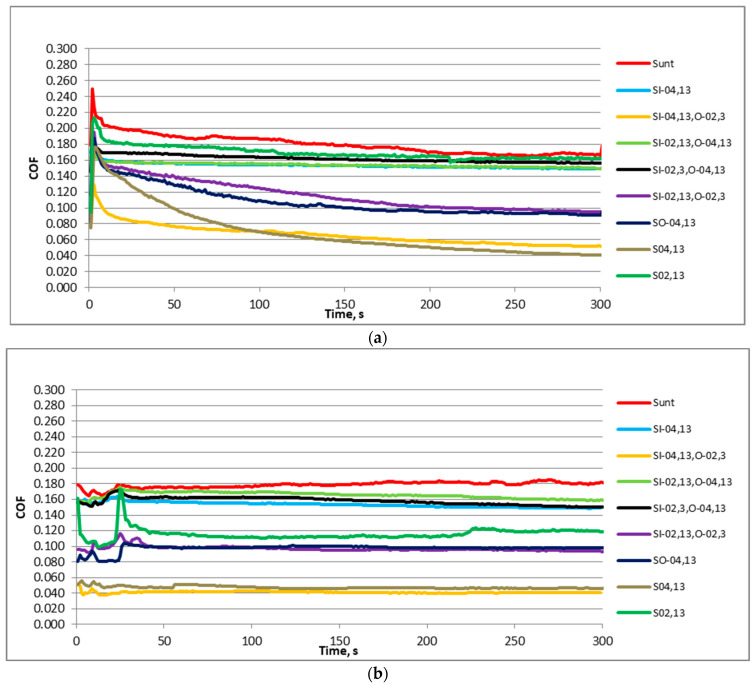
Representative courses of the coefficient of friction versus time for all sliding pairs for the normal force of 80 N and the frequency of displacement of 20 (**a**) and 40 Hz (**b**).

Figure 9 presents the representative courses of the coefficient of friction versus time for the oscillation frequencies of 20 Hz and 40 Hz and the normal load of 80 N of various tested sliding pairs. Generally, the initial shapes of the curves were similar to those shown in Figure 5. For a smaller frequency of displacement (Figure 9a), the friction coefficient increased sharply during the first 5 s. Then, the friction coefficients decreased. They were more stable than those shown in Figure 5a; after initial fluctuations, abrupt jumps in the friction did not occur.

Different shapes of the friction coefficient emerged with a higher frequency of oscillation (Figure 9b). The coefficient of friction increased during the first 30–35 s, then it decreased, then increased, and then obtained stable value. However, after initial fluctuations, the further changes in the friction coefficient were low. It seems that the friction coefficients were more stable, compared to those derive during the work at a smaller normal load (Figure 5). A high variability in the friction coefficient for the assembly with sample S_02,13_ occurred.

**Figure 10:**
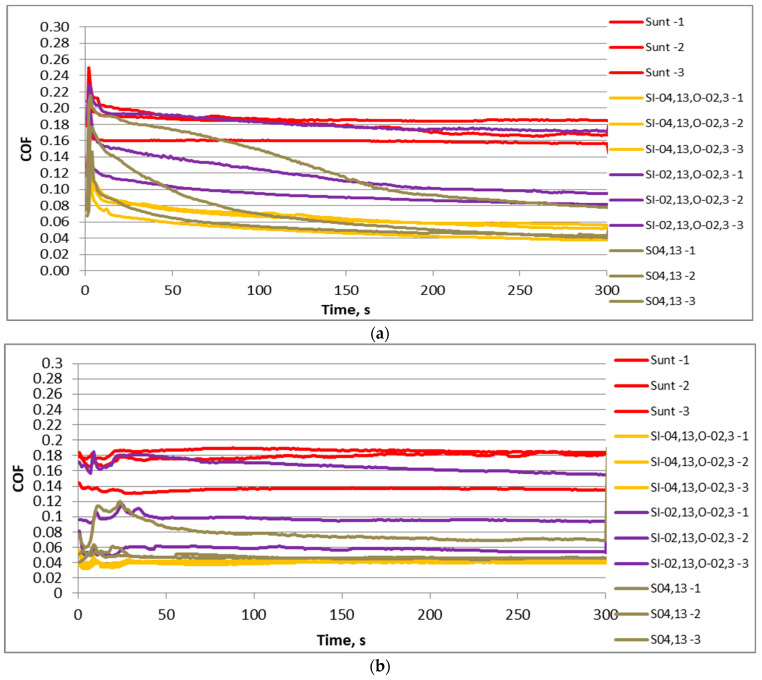
The coefficient of friction versus time for selected sliding pairs with samples Sunt, SI-02,13,O-02,3, SI-04,13,O-02,3 and S04,13 for the normal force of 80 N and the displacement frequency of 20 (**a**) and 40 Hz (**b**).

Figure 10 presents the friction coefficients versus time for three test repetitions of selected assemblies for the normal force of 80 N. Similarly to Figure 6, the lowest values and variations of the friction coefficient were reached for assemblies containing samples S_I-04,13,O-02,3_ and S_04,13_. The untextured sliding pair was characterized by the highest friction coefficient and a comparatively great friction variation. The sliding assembly with disc sample S_I-02,13,O-02,3_ caused the highest variability in the friction coefficient, while the values of the coefficient of friction were smaller than those of the untextured assembly, but higher than the sliding pairs with samples S_I-04,13,O-02,3_ and S_04,13_.

**Figure 11:**
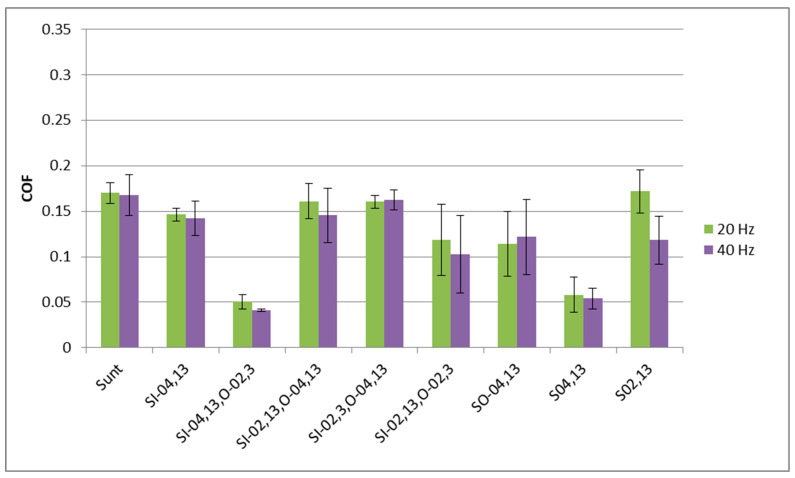
Mean values and error bars of the friction coefficients of different assemblies tested at the normal force of 80 N for the last 100 s.

Figure 11 presents the average values and error bars of the friction coefficient for assemblies tested at the normal force of 80 N for the last 100 s. When the frequency of displacement was smaller, the largest friction coefficient occurred for sample S_02,13_ followed by the untextured sample, discs S_i-02,13,0-04,13_ and sample S_I-04,13_. Smaller friction coefficients were obtained for samples S_I-02,13,0-02,3_ and S_0-04,13_; in these cases the variations of the friction coefficients due to test repetitions were large. The lowest friction coefficients were achieved for assemblies that contained the samples S_04,13_ and S_I-04,13,0-02,3_. The last mentioned sample corresponded to a low variation in the coefficient of friction. The highest friction reduction of 70% occurred for the S_I-04,13,0-02,3_ sample. 

For the normal load of 80 N and a frequency of displacement of 40 Hz, high coefficients of friction were acquired for assemblies with untextured samples and textured discs: S_I-0,02,O-04,13_ and S_I-04,13_. The S_0-04,13_, S_I-0,02,13,O-0,02-3_ and S_02,13_ samples manifested friction reduction; however, the variations in the friction coefficient due to test repetitions were comparatively high. In these cases, the maximum decrease in the coefficient of friction, compared to the untextured assembly, was 38%. A further decrease in the friction coefficient was reached for the disc sample S_04,13_. The smallest friction coefficient and the smallest fluctuation of friction due to test repetitions were achieved for the sliding pair with the disc sample S_I-04,13,0-02,3_—the friction reduction was approximately 80%, compared to the untextured sliding pair. In most cases, the increase in frequency of displacement caused a decrease in the friction coefficient.

**Figure 12:**
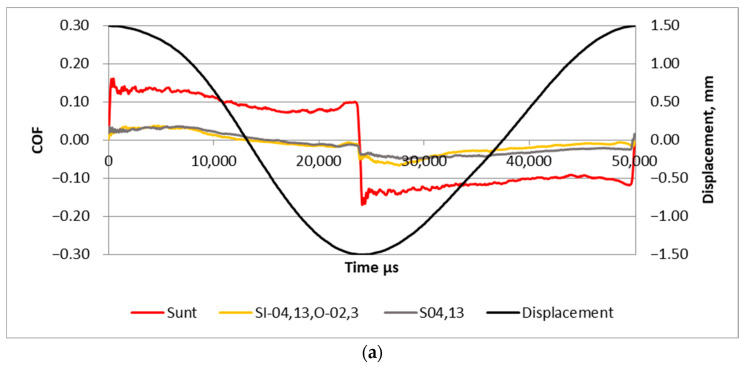

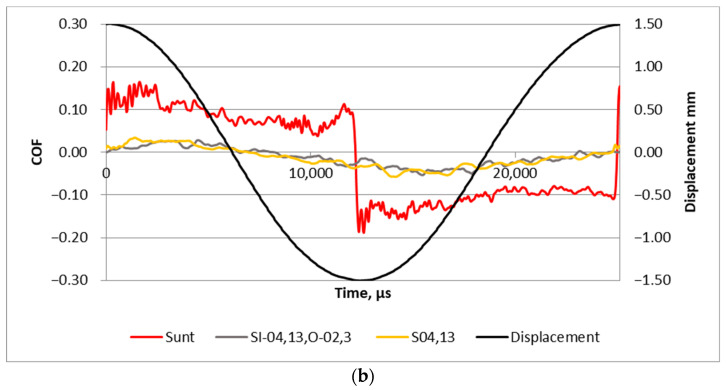
High-frequency analysis of the coefficients of friction obtained for untextured and textured samples for normal force of 80 N and displacement frequency of 20 (**a**) and 40 Hz (**b**).

Figure 12 presents examples of the friction coefficient within one stroke for assemblies with untextured S_unt_ and textured samples S_I-0,04,13,O-0,02,3_ and S_0,04,13_. The curves were similar to those presented in Figure 8. The smallest coefficients of friction were obtained for sliding pairs with textured samples. A higher frequency of displacement led to a higher fluctuation of the friction coefficient.

After the analysis of surfaces using scanning electron microscopy, it was found that the wear levels of the samples were small, and only the highest peaks were truncated (see also Figure 2 and Figure 4). The changes were greater when the friction forces were higher. The wear had an abrasive character with the presence of delamination.

**Figure 13:**
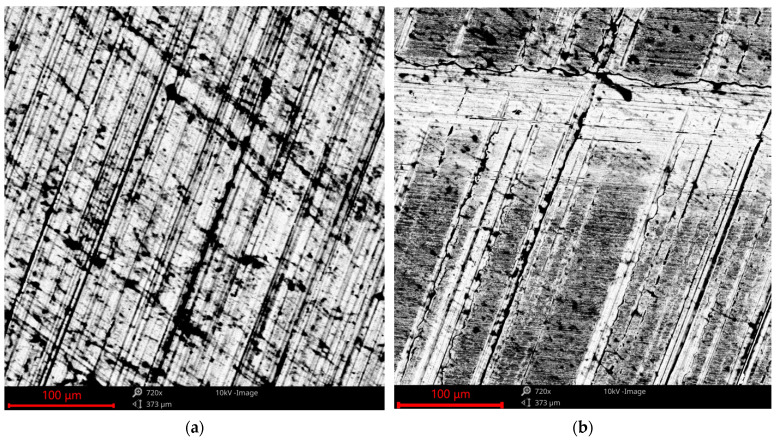
SEM images of the surface of the untextured sample before (**a**) and after the tribologic test (**b**).

Figure 13 shows images of an untextured surface before and after tribological tests. Due to wear, the surfaces became smoother, and the grooves resulting from initial grinding disappeared. 

**Figure 14:**
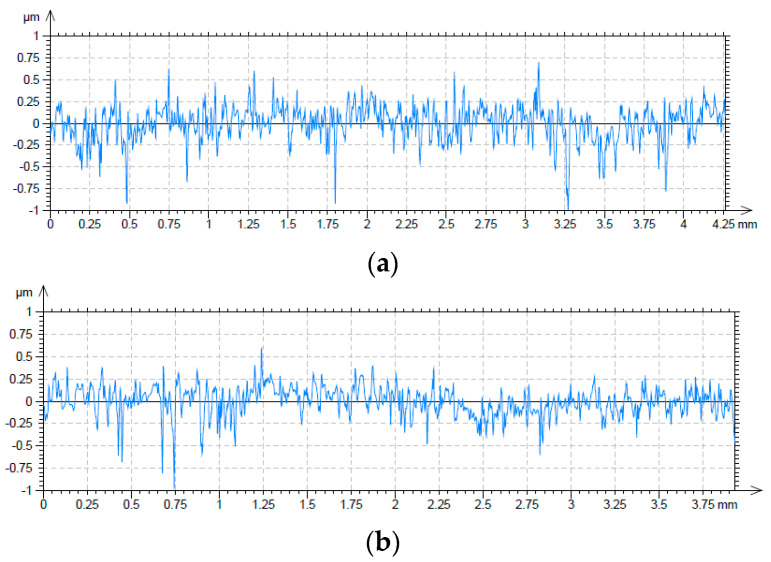
Roughness profiles of the surfaces of untextured samples before (**a**) and after the tribologic test (**b**).

Figure 14 presents roughness profiles of surfaces before (a) and after the tribological tests. During tests, the initial roughness was changed due to truncation of the roughness peaks. The roughness height Ra of untextured samples was 0.13 µm before and 0.1 µm after the test.

**Figure 15:**
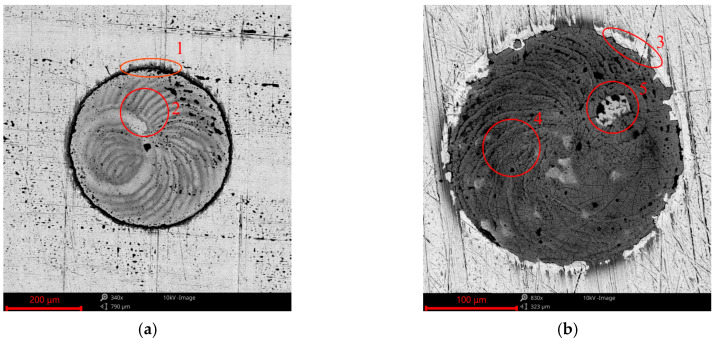
SEM images of the surface of the textured sample before (**a**) and after the tribologic test (**b**); with marked exemplary areas of 1—material flash, 2—bottom of oil pocket, 3—example area with removed material flash due to wear, 4—cracked oxidized bottom of oil pocket, 5—uncovered base material after detaching oxidized layer.

Figure 15 presents SEM images of the dimple before and after the tribologic test. A black ring is visible in Figure 15a (1—black places contain oxygen). This is a material flash created by an oxidized material, which was subjected to laser treatment. Elemental analysis performed using SEM showed that this black area (Figure 15a (1) contained 30–36% oxygen, 28–37% iron, and 30–41% carbon in weight concentration. At the bottom of the dimple, areas of oxidized material are also visible (Figure 15a (2). The bottoms of the oil pockets contained between 16 and 20% oxygen, 21 and 23% carbon and 58 and 60% iron in weight concentration. In the worn sample (Figure 14b), the material flash with oxides was destroyed by contact with the counter-sample, and the native material was opened (Figure 15b (3). In this area, the base material was uncovered and the weight concentration of oxygen was between 1 and 2%. In addition, at the bottom of the dimple, an oxidized material layer was visible (cracked area; Figure 15b (4). In some cases, this layer was detached from the surface (bright area, in which the native material was exposed; Figure 15b (5) due to friction. This phenomenon leads to the presence of wear debris in lubricating oil; however, its effect of increasing wear was negligible (wear was reduced due to the presence of oil pockets). 

The effect of surface texturing on the frictional resistance of sliding pairs depends on the dimple array and, to a smaller degree, on the frequency of displacement. Among the eight variants of surface texturing, five led to a reduction in friction for a smaller frequency of displacement, independently of the normal load. However, for a higher frequency of displacement, the beneficial effect of surface texturing was achieved in only three cases. This phenomenon was probably caused by higher fluctuations in the friction forces with a higher frequency of oscillation, both due to test repetitions and within one stroke. These fluctuations were caused by more stable working conditions with a smaller frequency of displacement.

Under a smaller frequency of displacement, partial surface texturing of S_I-0,04-13_ and S_O-0,04,13_ caused a reduction in friction compared to the untextured sliding pair. This reduction was greater for the S_O-0,04-13_ sample; however, it was accompanied by a higher friction variation. The effect of partial surface texturing was negligible for a higher frequency of oscillation of 40 Hz. It is evident from the literature review that oil pockets were created on the surface, working in reciprocating motion typically near reversal points. Only Urabe et al. [40] reduced the friction force by creating dimples in the middle zone of the cylinder liner. However, they applied shallow dimples with a high pit area ratio, differently from the oil pockets applied in this work. In cylinder liners, oil pockets are created near the dead center on the top to reduce oil consumption [43,44,45] and fuel consumption [44,45]. Oil consumption was not analyzed in this work. The formation of oil pockets in the middle part of the stroke is not recommended as the effect of surface texturing on fluid film lubrication is small [3]. However, in this work, mixed lubrication probably occurred in the middle part of the stroke when the sliding contact between untextured surfaces was studied. Partial surface texturing in various parts of the lower disc caused a decrease in friction coefficient. However, the beneficial effect of uniform surface texturing S_04,13_ was considerably greater than that of partial texturing. Ryk et al. [20,23] obtained opposing results related to piston rings. This difference was caused by different working conditions in this work and in [19,20].

The dimple pattern characterized by a diameter of 0.4 mm and a pit area ratio of 13% led to a notable decrease in friction compared to an untextured disc when dimples were created in both the middle part of the disc and near reversal points. This reduction was substantial for all working parameters (normal loads and displacement frequencies), and was the largest for a smaller load of 40 N and frequency of 20 Hz. This kind of surface texturing also caused small friction fluctuation as a result of test repetitions. The behavior of the sliding pair with disc sample S_04,13_ was better than that of sample S_02,13_, characterized by the same pit area ratio and a smaller dimple diameter. Sample S_02,13_ was characterized by twice the density of dimples of sample S_04,13_. The density of dimples means the number of dimples divided by the area of measurement (similarly to the density of peaks/summits). In technical literature, the density of oil pockets and the pit area ratio typically describe the ratio of the area of dimples to the measurement area. The disc sample S_04,13_ behaved better (friction coefficient value and variation) than sample S_02,13_ independently of normal loads and frequencies. Disc sample S_02,13_ corresponded to a high variation in the coefficient of friction within tribological tests, and due to test repetitions. Perhaps a larger diameter of the dimples in sample S_04,13_ led to better conditions generating hydrodynamic lift than in sample S_02,13_. 

The performance of the sliding pair with sample S_02,13_ was better than that of the untextured sample for a displacement frequency of 20 Hz and normal force of 40 N. In this case, the coefficient of friction of the sample S_02,13_ was also smaller than those of assemblies with discs with different dimple patterns in inner and outer disc zones: S_I-04,13,O-02,3_, S_I-04,13,O-02,13_, S_I-02,13,O-02,3_ and S_I-02,3O04,13_. Only the disc sample S_I-04,13,O-02,3_ led to a notable decrease in the friction coefficient, compared to the untextured assembly, for all frequencies and normal loads applied. In this case, the mean values of the friction coefficient were similar to that with the S_04,13_ sample, and the variations of the friction due to test repetitions were the smallest. These results prove that proper surface texturing in the middle (inner) part of the lower disc was of the highest significance. Perhaps in this zone, the possibility to create hydrodynamic lift as a result of the presence of oil pockets was large. 

Regarding the other sliding pairs, the disc sample S_I-02,13O-02,3_ led to a decrease in friction, especially under the higher normal load, of 80 N. Two other possibilities, S_I-02,13,O-04,13_ and S_I-02,3,O-04,13_, did not cause friction reduction. The results suggest that the dimple pattern of 0.4 mm diameter and 13% pit area ratio in the inner zone, and 0.2 mm diameter and 3% pit area ratio in the outer region, provided the best tribological performance. In the outer zone near the reversal points, the most difficult working conditions, and probably boundary friction, occurred. In this case a low pit area ratio is needed to ensure low unitary pressures (a high pit area ratio led to a growth in the unitary pressure). In the technical literature, the pit area ratio should be reduced in the central part of the liner [42,43,44,45]. However, different conditions were employed in this work and in the cylinder liner. In this research, the amount of lubricant was low, and it was not possible to obtain full lubrication in the middle part of the stroke, contrary to the behavior of the cylinder liner. The effect of surface texturing on oil consumption is typically analyzed in the cylinder liner, in contrast to this work. Zhu and Vladescu [38,39], respectively, also recommended a larger pit area ratio at the reversal points, compared to the central liner part. However, in these works, configurations similar to those of the piston-ring liner surface were tested. However, Hsu et al. [41] obtained different results. He found that narrow and deep dimples are recommended for mixed/boundary lubrication. These results are similar to those obtained in this research.

The study of the coefficient of friction within one stroke (Figure 8 and Figure 12) suggests that due to surface texturing, the oil film thickness was not interrupted at reversal points.

This analysis is restricted to the lubricated reciprocating sliding contact of a ring shape.

## 4. Conclusions

In this work, the effect of zonal surface texturing on friction reduction in lubricated reciprocating sliding during contact in a ring shape was studied. The experiment was carried out under various frequencies of displacement (20 and 40 Hz) and normal loads (40 and 80 N). The following conclusions have been reached:

1. Variable dimple patterns in different zones can lead to decreases in friction coefficient values and fluctuations. The best tribological performance was reached for a pit area ratio of 13% a diameter of 0.4 mm in the inner (central) sample zone and a pit area ratio of 3% and a diameter of 0.2 mm in the outer zones (near the reversal points). This sample led to low resistance to motion and the smallest friction variation from all tested sliding pairs. The dimple patterns in the inner zone determined the tribological behavior of the sliding pair;
2. With uniform texturing with a pit area ratio of 13%, the disc sample with a dimple diameter of 0.4 mm behaved better than that with a diameter of 0.2 mm, taking into consideration the reduction in the friction coefficient value and variation;
3. Partial surface texturing caused a reduction in the coefficient of friction at a frequency of displacement of 40 Hz and the normal load of 80 N. A reduction in friction due to surface texturing was not accompanied by a reduction in friction variation;
4. The effects of surface texturing on friction reduction were more visible for a frequency of displacement of 20 Hz, compared to 40 Hz;
5. The wear of disc samples was low; it depended mainly on the truncation of the highest surface peaks. Low coefficients of friction corresponded to smaller changes in surface topography due to wear.
6. Not only the pit area ratio, but also the density of dimples (defined as the ratio of the number of dimples to the area of measurement), are tribologically important parameters that characterize the dimple pattern.

## Figures and Tables

**Table 1:** Tested disc surfaces.

Surface Designation	Inner Zone	Outer Zones
S_unt_	T_unt_	T_unt_
S_I-04,13_	T_04,13_	Tunt
S_I-04,13,O-02,3_	T_04,13_	T_02,3_
S_I-02,13,0-04,13_	T_02,13_	T_04,13_
S_I-02,3,0-04,13_	T_02,3_	T_04,13_
S_I-02,13,0-02,3_	T_02,13_	T_02,3_
S_0-04,13_	T_unt_	T_04,13_
S_04,13_	T_04,13_	T_04,13_
S_02,13_	T_02,13_	T_02,13_

## Data Availability

The raw data supporting the conclusions of this article will be made available by the authors on request.
